# Preconditioning with cobalt chloride modifies pain perception in mice

**DOI:** 10.3892/etm.2015.2235

**Published:** 2015-01-29

**Authors:** TEODORA ALEXA, ANDREI LUCA, ANDREI DONDAS, CATALINA ROXANA BOHOTIN

**Affiliations:** Center for the Study and Therapy of Pain, ‘Grigore T. Popa’ University of Medicine and Pharmacy, Iaşi, Moldavia 70115, Romania

**Keywords:** analgesia, cobalt chloride, hyperalgesia, hypoxia, locomotor activity

## Abstract

Cobalt chloride (CoCl_2_) modifies mitochondrial permeability and has a hypoxic-mimetic effect; thus, the compound induces tolerance to ischemia and increases resistance to a number of injury types. The aim of the present study was to investigate the effects of CoCl_2_ hypoxic preconditioning for three weeks on thermonociception, somatic and visceral inflammatory pain, locomotor activity and coordination in mice. A significant pronociceptive effect was observed in the hot plate and tail flick tests after one and two weeks of CoCl_2_ administration, respectively (P<0.001). Thermal hyperalgesia (Plantar test) was present in the first week, but recovered by the end of the experiment. Contrary to the hyperalgesic effect on thermonociception, CoCl_2_ hypoxic preconditioning decreased the time spent grooming the affected area in the second phase of the formalin test on the orofacial and paw models. The first phase of formalin-induced pain and the writhing test were not affected by CoCl_2_ preconditioning. Thus, the present study demonstrated that CoCl_2_ preconditioning has a dual effect on pain, and these effects should be taken into account along with the better-known neuro-, cardio- and renoprotective effects of CoCl_2_.

## Introduction

Cobalt (Co) is a ferromagnetic transition metal, essential to human health, that plays a critical role in the synthesis of vitamin B_12_. The toxic effect of Co was first described in 1966, when beer drinkers developed a cardiomyopathy characterized by pericardial effusion, elevated hemoglobin concentrations and congestive heart failure, due to the addition of Co sulfate to the beer as a stabilizer (1).

The mechanism by which Co acts on cells is controversial. Previous studies have demonstrated that Co modifies mitochondrial permeability by opening the transition pores, which leads to mitochondrial swelling and electrical membrane potential collapse (2), and inhibits crucial enzymes that participate in the mitochondrial respiratory chain due to its high affinity for sulfhydryl groups (3). In addition, Co has been demonstrated to decrease neurotransmitter-induced postsynaptic responses by inhibiting synaptic transmission through the presynaptic blockage of calcium (Ca^2+^) channels (4). Co has also been hypothesized to function as a Ca^2+^ channel antagonist, competing for intracellular Ca^2+^-binding proteins and thus exerting inhibitory effects on Ca^2+^ signaling (5). Furthermore, Co administration in rats has been shown to induce a depletion of neurotransmitters, including dopamine, norepinephrine and serotonin, which suggests that Co can cause memory impairment (6).

A previous study indicated that Co chloride (CoCl_2_) activates hypoxia-inducible factor (HIF)-1α, acting as a hypoxia-mimetic and inducing reactive oxygen species (ROS)-mediated toxicity (7). Furthermore, CoCl_2_ is able to stabilize HIF-1α, a key determinant of the cellular response to hypoxia, which has made the compound one of the most commonly used hypoxia-mimetic agents (8). Previous animal studies have demonstrated that CoCl_2_ preconditioning increases mitochondrial biogenesis, glucose uptake and metabolism in the skeletal muscles (9), and attenuates vascular leakage and ROS-induced hypoxia generation in the brain (10). Research indicates that CoCl_2_ preconditioning has cardioprotective (11), renoprotective (12) and neuroprotective effects (13).

There is increasing evidence that superoxide, a member of the ROS family produced during hypoxia, and mitochondrial activation, are involved in the development of chronic pain, in the transition from acute to chronic pain, in opiate-induced hyperalgesia and in antinociceptive tolerance (14).

The aim of the present study was to investigate the effects of modulating mitochondrial function and cellular antioxidant capacity by CoCl_2_ preconditioning on nociception and locomotor activity in mice.

## Materials and methods

### Animals

Experiments were conducted on 80 male BALB/c mice (weight, 28–34 g), housed at 21±2°C under a 12-h light/dark cycle, with access to food and water *ad libitum*. The study was conducted in accordance with the European Communities Council Directive 2010/63/EU (15), the ‘Guidelines for the use of animals in research (1991)’ (16) and the Grigore T. Popa University of Medicine and Pharmacy (Iaşi, Romania) ethical guidelines for the experimental investigation of pain in conscious animals.

### Drugs

CoCl_2_, glacial acetic acid and formaldehyde solution (37 wt% in H_2_O) were obtained from Sigma-Aldrich (St. Louis, MO, USA). The compound was freshly diluted in a saline solution (0.9% NaCl; B. Braun Melsungen AG, Melsungen, Germany) and was administered via an intraperitoneal (i.p.) route, with doses expressed as mg per kg of body weight (mg/kg b.w.).

### Tests and models of nociception and pain

#### Hot-plate test (HPT)

The HPT was performed on 12 mice in the CoCl_2_ group and 10 mice in the saline group, as previously described (17). The animals were placed in an open Plexiglas tube on a hot-plate device (model-DS 37; Ugo Basile Srl, Varese, Italy) at a temperature of 55±0.1°C. The time between the placing of the animal and the occurrence of licking, shaking of hind paws or jumping off the surface was recorded as a response latency. The experiment cut-off time was set to 15 sec to prevent tissue damage.

#### Tail-flick test (TFT)

A TFT was performed on 12 mice in the CoCl_2_ group and 10 mice in the saline group (18). The distal portion of each mouse tail was placed on the heat source of the apparatus (Tail Flick Unit-37360; Ugo Basile Srl) and the time until the animal removed its tail was defined as the tail-flick latency, with a 12-sec cut-off time.

#### Plantar test

A Plantar test using the Hargreaves method was applied for 12 mice in the CoCl_2_ group and nine mice in the saline group (19). The mice were placed into clear acrylic boxes on a Plexiglas floor, and the radiant heat source from the Hargreaves unit (Plantar Test-37370; Ugo Basile Srl) was placed under the hind paw. The thermal withdrawal threshold was recorded as the time between the start of the radiant heat stimulus and the withdrawal or licking of the hind paw, with a 20-sec cut-off time. The mean paw withdrawal latency was obtained from the average of three separate trials.

#### Mechanical sensitivity test

The mechanical withdrawal threshold was measured using an automatic Von Frey method with a Dynamic Plantar Aesthesiometer 37450 (Ugo Basile Srl). A total of 12 mice in the CoCl_2_ group and nine mice in the saline group were placed into acrylic chambers with wire mesh floors. A servo-controlled mechanical stimulus was applied repeatedly and alternately to the plantar surface of each hind paw. The time elapsed until the pressure exerted by the filament evoked a clear voluntary hind paw withdrawal response was automatically recorded. To yield the mean value, mechanical withdrawal thresholds were measured in triplicate for each animal.

For the aforementioned tests, hyperalgesia was quantified as the percentage decrease in the withdrawal threshold: (Baseline value - CoCl_2_ value) × 100/baseline value (20).

#### Writhing test (WT)

A WT was performed to measure visceral pain, as described by Koster *et al* (21). After becoming accustomed to the acrylic chambers, the mice (eight in the CoCl_2_ group and eight in the saline group) received i.p. injections of 1.0% (v/v) acetic acid (0.1 ml/10 mg b.w.). The number of abdominal writhes was counted over a period of 30 min.

#### Paw formalin test (PFT) and orofacial formalin test (OFT)

These tests consisted of injecting 20 μl formalin (5%) subcutaneously into the plantar surface of the right hind paw (PFT) or the right whisker pad (OFT). The total time (sec) that the mice (n=10 in the CoCl_2_ and saline groups for PFT and OFT) spent licking and/or biting the injected paw/whisker during the first/neurogenic phase (PFT, 0–9 min; OFT, 0–6 min) and the second/inflammatory phase (PFT, 10–40 min; OFT, 7–40 min) were recorded (22,23).

For formalin- and acetic acid-induced pain, the antinociceptive activity was expressed as the percentage inhibition of nociceptive behavior (INB), using the following formula: %INB = (mean saline group - mean CoCl_2_ group) × 100/(mean saline group).

### Study design

The 80 mice were divided into two groups. The CoCl_2_ group mice (n=30) received 12.5 mg/kg b.w. CoCl_2_ (i.p.) daily for 21 days, while the saline group mice (n=50) received an equivalent volume of saline. The dose of 12.5 mg/kg b.w. was selected in accordance with published data concerning CoCl_2_ preconditioning (10,24).

A total of 12 out of the 30 mice from the CoCl_2_ group underwent testing prior to CoCl_2_ administration (baseline) and thereafter each day for the TFT and HPT, and weekly for the mechanical and thermal hyperalgesia tests. All the tests were performed prior to the daily CoCl_2_ injection. The other 18 mice from the 30 CoCl_2_ mice received CoCl_2_ daily for 21 days. During CoCl_2_ preconditioning two mice died. The remaining 28 mice were divided as follows: 8 underwent the WT, 10 mice underwent PDT, and 10 mice underwent OFT. The control group received an equivalent volume of saline daily. A total of 10 mice out of the 50 mice from the control group underwent baseline testing for the TF and HPT, and another 10 mice were tested for mechanical and thermal hyperalgesia. Throughout the experiment, three mice from the control group died, therefore the number of mice tested for thermal hyperalgesia was nine, and the number of mice included in the WT was eight. Data from the tests performed at baseline and days 7, 14 and 21 are presented for both the CoCl_2_ and saline groups. In addition, the WT, OFT and PFT were performed 24 h after the last dose of CoCl_2_ and compared with the saline group.

### Statistical analysis

Data are expressed as the mean ± standard error of the mean. The following values are presented in the results: *F* ratio and the number of degrees of freedom, outcome and sigificance value. Statistical evaluations were performed using SPSS v20.0 software (IBM, Armonk, NY, USA). An unpaired Student’s t-test and repeated analysis of variance (ANOVA), followed by the Bonferroni post hoc test, were used when appropriate. P<0.05 was considered to indicate a statistically significant difference.

## Results

### TFT

**Figure 1:**
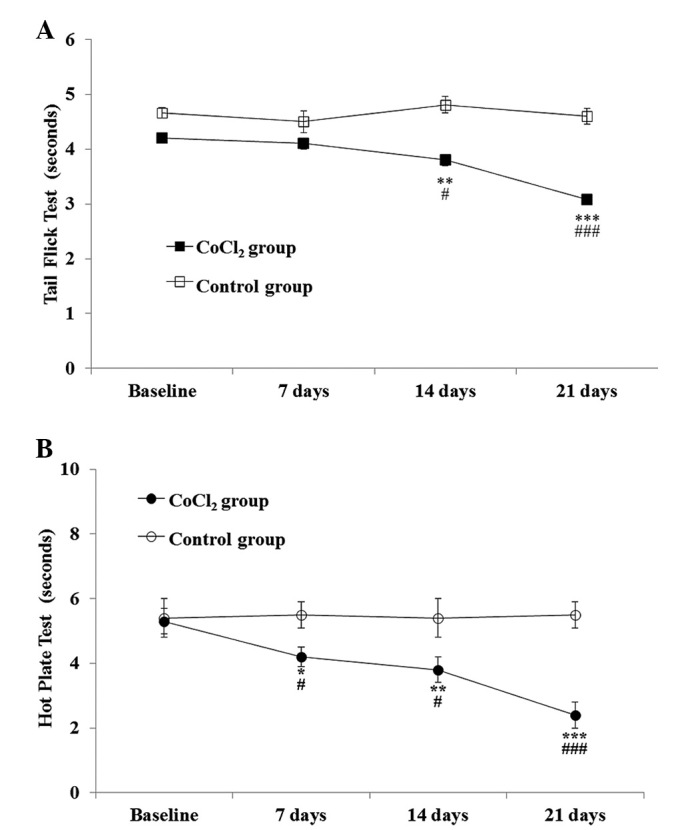
(A) Tail flick and (B) hot plate latencies over time (baseline and days 7, 14 and 21) in mice preconditioned with CoCl_2_ and in the saline group. Values are represented as the mean ± standard error of the mean. ^*^P<0.05, ^**^P<0.01 and ^***^P<0.001, vs. baseline; ^#^P<0.05 and ^###^P<0.001, vs. saline group. CoCl_2_, cobalt chloride.

CoCl_2_ preconditioning was shown to have a statistically significant effect on the TFT when compared with saline administration [F(1,20)=52.7; P<0.001], and this effect became more pronounced over time [F(3,33)=10.9; P<0.001], as assessed by repeated measures ANOVA. The Student’s t-test for independent samples showed a statistically significant lower tolerance for thermal stimuli in the CoCl_2_ group, which was observed in the second week (P=0.01) and persisted until the end of the experiment (day 21; P<0.001). The decrease in the withdrawal threshold ranged between 8.52±3.8% at week one and 25.56±3.8% at week three (Fig. 1).

### HPT

With regard to the HPT, repeated measures ANOVA indicated that CoCl_2_ preconditioning had a statistically significant effect on the response latency when compared with the saline group [F(1,20)=17.08; P=0.001]. The effect became more pronounced over time [F(3,33)=12.7; P<0.001].

Statistically significant differences were observed between the saline and CoCl_2_ groups one week after CoCl_2_ administration (P=0.03). By the end of the second week, HPT latencies significantly decreased (P=0.03, vs. saline group and P=0.01, vs. baseline) and remained lower than the baseline values until the end of the experiment (day 21; P<0.001, vs. saline group and baseline). By the final day of the experiment, the decrease in the withdrawal threshold was 49.35±5.64% (Fig. 1).

### WT

A similar number of writhes were recorded over a 30-min period in the CoCl_2_ group when compared with the saline group (P=0.1).

### PFT

**Figure 2:**
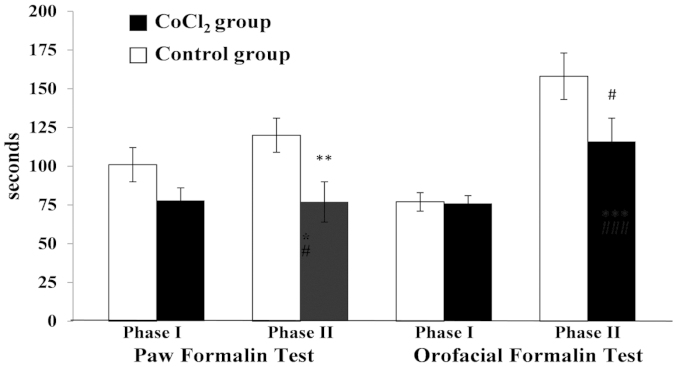
Effect of CoCl_2_ preconditioning (filled bars) on orofacial and plantar formalin-induced pain, as compared with the saline-injected (open bars) group. Values are represented as the mean ± standard error of the mean. ^**^P=0.01 and ^#^P=0.06, vs. saline group. CoCl_2_, cobalt chloride.

No statistically significant differences were observed between the CoCl_2_ and saline group mice for the first phase (P=0.1); however, the CoCl_2_ group mice spent 23.2% less time grooming the affected paw when compared with the saline group. In the second phase of the PFT, the CoCl_2_ group mice spent significantly less time grooming/licking the affected paw when compared with the saline group, with an INB of 35.7% (P=0.01; Fig. 2).

### OFT

Pain behavior did not statistically differ between the two groups in the two phases of the test (P=0.8, first phase; P=0.06, second phase). However, the INB for the CoCl_2_ group mice in the second phase was 27.08% (Fig. 2).

### Plantar test

**Figure 3:**
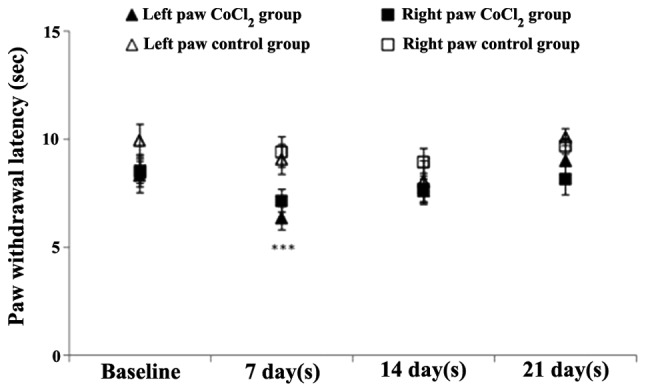
Thermal hyperalgesia was assessed using the Hargreaves method and expressed as the paw withdrawal threshold (sec) in mice treated with CoCl_2_ and saline. Values are represented as the mean ± standard error of the mean. ^***^P<0.001, vs. saline group. CoCl_2_, cobalt chloride.

In the preconditioned mice, the CoCl_2_ had a statistically significant effect [F(1,40)=7.3; P=0.01], while time had a marginal effect [F(2.08,48.01)=2.5; P=0.08]. At the end of the first week, a significant thermal withdrawal threshold difference was observed in the CoCl_2_ group when compared with the saline group (P=0.01). However, these changes returned to baseline values in week three (8.19±0.5 sec at baseline vs. 8.57±0.6 sec at week three; P=0.6). Throughout the experiment, no statistically significant differences were observed in the CoCl_2_ group when compared with the baseline values (Fig. 3).

### Mechanical sensitivity test

Repeated measures ANOVA indicated that CoCl_2_ preconditioning had a statistically significant effect on the mechanical sensitivity test when compared with saline administration [F(1,40)=27.9; P<0.001]. In addition, this effect became more pronounced over time [F(3,69)=9.76; P<0.001].

**Figure 4:**
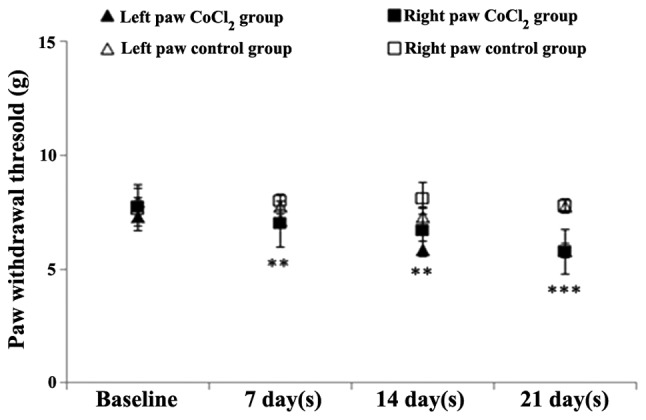
Mechanical allodynia was assessed using the Von Frey method, and expressed as the paw withdrawal threshold (g) in CoCl_2_ preconditioned mice and the saline group. Values are represented as the mean ± standard error of the mean. ^**^P<0.01 and ^***^P<0.001, vs. saline group. CoCl_2_, cobalt chloride.

Throughout the experiment, the mechanical withdrawal thresholds progressively decreased in the CoCl_2_ group. In the first week, a statistically significant difference was observed in the CoCl_2_ group when compared with the saline group (P=0.01), and in the second week when compared with the CoCl_2_ baseline levels (P=0.001). At the end of the experiment, the mechanical withdrawal threshold was 20% lower than the baseline value of the CoCl_2_ group, and the difference with the saline group was statistically significant (P<0.0001; Fig. 4).

## Discussion

The present study examined the effects of hypoxic preconditioning on nociception and pain. The results indicated that CoCl_2_ hypoxic preconditioning resulted in a pronociceptive effect on the HPT and TFT after one and two weeks of daily administration, respectively. These pronociceptive effects persisted until day 21 of treatment. Mechanical allodynia was similar to the HPT. After the first week, thermal hyperalgesia (Plantar test) was observed; however, this change was temporary and by the end of the experiment the mean thermal withdrawal thresholds had returned to baseline values. In addition, CoCl_2_ preconditioning was shown to modify pain perception in the second phase of formalin-induced pain; however, no effects were observed on visceral and acute formalin pain.

Previous studies have indicated that the TFT is affected by stimuli that act primarily at a spinal level (25), whereas the HPT reflects supraspinal sensory integration (26). By comparing the time frame for these two tests, the results of the present study indicate that supraspinal pain structures are affected one week following CoCl_2_ administration, whereas spinal structures are affected after two weeks. Once established, these changes persisted throughout the duration of the experiment. These data are consistent with the literature, showing that spinal somatosensory-evoked potentials are more resistant to severe hypoxia than cortical somatosensory-evoked potentials (27). In addition, the data indicate that the spinal nociceptive reflexes (TFT) are more resistant to CoCl_2_ hypoxic preconditioning than supraspinal responses to nociception (HPT).

Mechanical allodynia appeared in the first week of the study and persisted until the last day of the experiment, while thermal hyperalgesia returned to baseline levels in the second week. Previous studies have also reported behavioral differences between mechanical and thermal hyperalgesia (28–30). Thermal hyperalgesia appears to be dependent on opioid-sensitive small-diameter primary afferent fibers, while mechanical allodynia is considered to rely on a largely independent small-fiber input. In addition to the differences in neuroanatomical pathways, changes in 5-hydroxytryptamine (29), opioid (30), nitric oxide/cGMP/ATP-sensitive K^+^ channels (31) or in ATP distribution along the peripheral and central nervous system have been considered as alternative explanations for the behavioral differences. The results of the present study, with regard to thermal and mechanical hyperalgesia, suggest a different response of A and C fibers to hypoxia.

Apostoli *et al* hypothesized that Co may have a specific neurotropism; therefore, CoCl_2_ may have a neurotoxic effect (32). In addition, chronic occupational exposure to Co has been shown to induce sensory-motor polyneuropathy in humans (33), while in animal models, Co has been shown to have neurotoxic effects *in vitro* and *in vivo* (34).

Contrary to the hyperalgesic effect on thermal pain, CoCl_2_ preconditioning decreased the time spent grooming the affected area in the inflammatory phase of formalin-induced pain in the paw and orofacial models. However, no effect on the nociceptive phase of the formalin tests was observed.

Hypoxic preconditioning has been documented to protect the brain (and other tissues) from ischemic insults and increase resistance to other types of injury. However, hypoxia causes a rapid reduction in intracellular ATP and a delayed increase in extracellular purine levels (35). Thus, the adenosine released secondary to hypoxia may act on the P2Y (a protein G-coupled receptor) and P2X (a ligand-gated cation channel receptor) adenosine receptors, which have different effects according to the concentration of the receptor along the nervous system and the ATP levels in the synapses or nervous cells (36).

Previous studies have suggested that the sensory experience of pain depends on descending pain modulator circuits arising from the rostral ventromedial medulla (RVM) (37). Thus, nociception is the consequence of enhancement (ON cells-pain facilitatory cells) or inhibition (OFF cells-pain inhibitory cells) of the spinal dorsal horn neurons by the RVM projection (38).

In formalin-induced pain, adenosine injected into the periaqueductal gray region has been shown to produce antinociceptive effects as a consequence of its ability to act on the ON and OFF cells from the RVM (39). By contrast, in the TFT and HPT, the ON and OFF cells were shown to be activated only during motor reactions, indicating that the RVM does not play an important role in the latencies observed in these tests. Therefore, it was hypothesized that the RVM may be one of the nervous system structures involved in the analgesic effect of CoCl_2_ hypoxic preconditioning.

The results obtained in the formalin-induced pain models are unlikely to be a consequence of motor disturbance, since locomotor activity and coordination did not exhibit statistically significant differences between the CoCl_2_ and saline groups (data not shown).

The results of the present study indicate that CoCl_2_ hypoxic preconditioning may have a neurotoxic effect, with observations of pronociception on thermal nociception, increased mechanical hyperalgesia and diminished pain sensibility on formalin-induced persistent pain. These effects may result from the capacity of CoCl_2_ to inhibit mitochondrial function and modulate ROS. Furthermore, an increased tolerance to pain was observed in the second phase of the formalin-induced pain models. Thus, in addition to a possible Co-induced neuropathy, a centrally-mediated analgesic effect of hypoxic preconditioning cannot be excluded.

In conclusion, the present study demonstrated that CoCl_2_ hypoxic preconditioning is a multifaceted phenomenon, and a balance between beneficial and detrimental effects must be taken into account in future experimental studies.
